# Barriers to successful implementation of care in home haemodialysis (BASIC-HHD):1. Study design, methods and rationale

**DOI:** 10.1186/1471-2369-14-197

**Published:** 2013-09-17

**Authors:** Anuradha Jayanti, Alison J Wearden, Julie Morris, Paul Brenchley, Inger Abma, Steffen Bayer, James Barlow, Sandip Mitra

**Affiliations:** 1 Department of Nephrology, Manchester Royal Infirmary, Manchester M13 9WL, UK; 2 School of Psychological Sciences, University of Manchester, Manchester M13 9PL, UK; 3 Department of Biostatistics, University of Manchester, Manchester M23 9LT, UK; 4 HaCIRIC, Imperial College Business School, London SW7 2AZ, UK

**Keywords:** Barriers, Home haemodialysis, Mixed methods, Qualitative, Organisation, Adoption, Quality of life

## Abstract

**Background:**

Ten years on from the National Institute of Health and Clinical Excellence’ technology appraisal guideline on haemodialysis in 2002; the clinical community is yet to rise to the challenge of providing home haemodialysis (HHD) to 10-15% of the dialysis cohort. The renal registry report, suggests underutilization of a treatment type that has had a lot of research interest and several publications worldwide on its apparent benefit for both physical and mental health of patients. An understanding of the drivers to introducing and sustaining the modality, from organizational, economic, clinical and patient perspectives is fundamental to realizing the full benefits of the therapy with the potential to provide evidence base for effective care models. Through the BASIC-HHD study, we seek to understand the clinical, patient and carer related psychosocial, economic and organisational determinants of successful uptake and maintenance of home haemodialysis and thereby, engage all major stakeholders in the process.

**Design and methods:**

We have adopted an integrated mixed methodology (convergent, parallel design) for this study. The study arms include a. patient; b. organization; c. carer and d. economic evaluation. The three patient study cohorts (n = 500) include pre-dialysis patients (200), hospital haemodialysis (200) and home haemodialysis patients (100) from geographically distinct NHS sites, across the country and with variable prevalence of home haemodialysis. The pre-dialysis patients will also be prospectively followed up for a period of 12 months from study entry to understand their journey to renal replacement therapy and subsequently, before and after studies will be carried out for a select few who do commence dialysis in the study period. The process will entail quantitative methods and ethnographic interviews of all groups in the study. Data collection will involve clinical and biomarkers, psychosocial quantitative assessments and neuropsychometric tests in patients. Organizational attitudes and dialysis unit practices will be studied together with perceptions of healthcare providers on provision of home HD. Economic evaluation of home and hospital haemodialysis practices will also be undertaken and we will apply scenario ("what … if") analysis using system dynamics modeling to investigate the impact of different policy choices and financial models on dialysis technology adoption, care pathways and costs. Less attention is often given to the patient’s carers who provide informal support, often of a complex nature to patients afflicted by chronic ailments such as end stage kidney disease. Engaging the carers is fundamental to realizing the full benefits of a complex, home-based intervention and a qualitative study of the carers will be undertaken to elicit their fears, concerns and perception of home HD before and after patient’s commencement of the treatment. The data sets will be analysed independently and the findings will be mixed at the stage of interpretation to form a coherent message that will be informing practice in the future.

**Discussion:**

The BASIC-HHD study is designed to assemble pivotal information on dialysis modality choice and uptake, investigating users, care-givers and care delivery processes and study their variation in a multi-layered analytical approach within a single health care system. The study results would define modality specific service and patient pathway redesign.

**Study Registration:**

This study has been reviewed and approved by the Greater Manchester West Health Research Authority National Research Ethics Service (NRES) The study is on the NIHR (CLRN) portfolio.

## Background

### Burden of ESRD

Chronic kidney disease (CKD) is a global public health concern ([1,2]). The tip of the CKD ‘iceberg’ is manifest in end stage renal disease (ESRD), when, to sustain life, some mode of renal replacement therapy (RRT) becomes necessary. Typically, there are three options available to patients who would like to consider treatment. These are transplantation (TX), home-based dialysis (home haemodialysis-HHD and peritoneal dialysis-PD) or hospital-based/In-Centre haemodialysis (ICHD). The global explosion in uptake of dialysis in the 80s and 90s, due to lack of availability of donor organs to meet demands for transplantation, is a testimony to the success of dialysis technology. However, patient outcomes on dialysis have been poor, and in this context, extended haemodialysis at home has delivered the best results.

Management of ESRD with haemodialysis began in the 1960s. The use of HHD modality was at its peak in the early 1980s (up to 2,200 patients), representing 61% of haemodialysis patients. Hospital dialysis units expanded across the UK in the 80s and 90s and ‘satellite’ hospital units emerged trying to meet the demand - as they were able to accommodate the ever increasing numbers of patients needing long term dialysis therapy. High rates of attrition from home HD were noted in the 90s. The prevalence of HHD modality dropped, to its usage in just 445 HD patients (2.4%) in 2006 [3]. The evolution of dialysis therapies has resulted in a gradual diminution of home based therapies to be largely replaced by ICHD.

This phenomenon has posed a challenge to clinicians, service providers and policymakers as scientific evidence in recent times has proposed a strong argument for greater adoption of this modality to improve patient outcomes on dialysis. The optimal uptake of HHD modality with current technology remains unknown. The case for HHD is made from the evidence of its benefits on clinical outcomes (better cardiovascular health, haemoglobin, blood pressure control, medication burden, sleep, nutritional status, fewer hospitalisations and better quality of life) compared to facility-based haemodialysis. Furthermore, published data have demonstrated extremely high technique survival rates (hence sustainability) in HHD [4].

Implementing home based therapies aligns closely with the government initiative of providing care ‘closer to home’. The Department of Health in the UK carried out an extensive review of self-care support, encompassing large numbers of systematic reviews, observations and surveys in a wide variety of clinical conditions and found clear evidence of beneficial health outcomes for patients and better use of health and social care resources [5]. Studies support cost-effectiveness of HHD when compared to ICHD. In one systematic review of 27 studies undertaken between 1978 and 2001, eighteen of these considered cost effectiveness and showed lower costs associated with HHD [6]. Even switching from hospital-based to home-based haemodialysis would optimise cost effectiveness [7]. More appropriate economic analysis will require other considerations, such as costs associated with home conversions, travel reduction, return to work and contribution thereby to the economy. The broader societal economic benefits of home haemodialysis include better full time employment of home and nocturnal dialysis patients [8]. Many dialysis units operate contracts with different funding sources and there are several cost variables which need to be considered.

### Adoption barriers to home haemodialysis

Variation in the uptake and prevalence of HHD is a worldwide phenomenon. Different health care systems, variable practice and reimbursement models have been implicated. Demographics, service provision landscape and social attitudes have evolved over time. Comorbidities, such as diabetes, have been on the rise (32.7% diabetics starting RRT in 2010). Within the last decade, there has been a resurgence of interest in HHD. This may account for the small increase in the proportion of patients receiving haemodialysis in their own homes since 2006 (up to 3.4% in 2010) [3]. This however, falls far short of the NICE guidance. Interestingly, despite national policy, NICE guidance, several initiatives and interventions within a single health care system (NHS), UK registry data suggest an atlas of variation in the proportion of dialysis patients receiving home HD (0% in 13 centres, to >5% in 8 centres) [3].

In the last decade, very few studies have been done to understand this phenomenon nationally and internationally. Studies from several countries have contributed to the understanding of some reasons for why the rate of adoption of home therapies in general (PD & HHD), may be slow. Earlier studies [9] failed to show any association between centre and patient demographics to modality prevalence. An association study between social deprivation and survival on RRT in England and Wales between 1997 and 2004, found inequitable access to RRT of individuals from deprived areas, using the Townsend index [10]. Patients who presented within 3 months of requiring dialysis were less likely to receive a home dialysis treatment in a survey by Lamiere et al. [11]. The impact of therapy specific patient education on choice is highlighted in several studies and does impact on home-based dialysis therapies– although these studies have mainly focused on PD [12]. The quality and duration of pre-dialysis education and the level of support, in the form of a team of specialist nurses, may have an influence on the number of patients choosing a home modality [13,14]. A systematic study of the barriers to uptake of home dialysis, in the USA [15], highlighted ‘current under-usage of home dialysis and identified problem areas including, limited and unmandated home dialysis training of nephrology fellows, lack of synchronised education of ESRD care providers, Medicaid services’ poor reimbursement policies which dis-incentivises home based therapy’. In an Australian survey [16], the most commonly reported impediments to expanding home dialysis services were operational and infrastructural factors such financial disadvantage for home HD patients, and lack of physical infrastructure for training, support and education. Areas of concern for expanding home dialysis programmes included psychiatry support, access to respite care and home visits, and lack of support from medical administration and the government. Clinician’s bias to one or the other modality and poor exposure to PD during training or recent completion of training were found be associated with bias against home dialysis therapies in general [17-20]. A different survey investigated reasons behind prevalent in-centre haemodialysis patients, choosing not to perform self-care dialysis. The outcomes suggest that ‘human factors such as, fear of change in general, fear of social isolation, not being prepared to stay awake on dialysis, time constraints preventing self-care, needle phobia and fear of reduced interaction were associated with a negative attitude towards self-care dialysis’ [21].

Many surveys generally indicate the widespread belief of physicians and care providers of the benefit of HHD which is in sharp contrast to the practice of this modality in their units. A survey by the Renal Registry demonstrated a broad range of opinions about dialysis modality related survival and quality of life reasons held by UK nephrologists [3]. In a large opinion based survey over 7000 nephrology health-care professionals were given questionnaires at five major international dialysis conferences in 2006 [22]. This survey identified patient motivation as one of the strongest drivers of self-care dialysis at home. The need for dedicated resources for staff to devote time to developing such motivation is given as one of the major reasons for the slow adoption. Under ideal conditions, it is felt that one-third of all patients starting dialysis can be trained to perform self-care dialysis.

### Limitations of published work

Most published analyses on adoption barriers to home haemodialysis from the UK, have been limited by examining factors in isolation, or from studies based on opinions, questionnaires and surveys which lack consensus. Many such surveys and studies are limited by gathering views of home haemodialysis enthusiasts. Registry data collection is limited to the clinical dataset and does not incorporate delivery aspects, patient reported outcomes and factors that define treatment preference and pathways of this modality. Investigating centres with variable practice and uptake (high, low or absent) simultaneously can potentially eliminate bias and provide more complementary and valid datasets for comprehensive analysis and interpretation. There is published literature of interview-based studies involving small numbers of home and hospital based HD patients. Whilst they highlight the perceived problems with home HD procedure, they have not been solution seeking and the choice of patients for such qualitative studies, has not been systematic. Besides, studies have not captured the journey of the predialysis patient in the months preceding the start of dialysis, when the crucial decision making process is initiated. It is also apparent that very few studies have a focus on the views of predialysis patients in preparation for commencement of renal replacement therapy, and in those where they have been included, HHD may not have been one of the modality choices on offer. Given the complexity of the decision-making process, further work with sufficient patient numbers is needed, to fully understand the nuances specific to home haemodialysis. In addition to patient clinical factors influencing their psychosocial state and choice of modality, a greater understanding of the complex interplay of patient and organisational factors and their impact on the adoption of home haemodialysis therapy is not available at the present time.

### Study objectives

The primary objective of the BASIC-HHD study is to conduct a comprehensive and systematic study of the barriers to and enablers of successful uptake and maintenance of HHD across multiple centres with low, medium and high prevalence rates of home HD. Care pathways of predialysis, incident and prevalent dialysis patients will also be investigated under clinical, psychosocial and organisational domains.

Additionally, the secondary objectives are, to

a) Investigate biomarkers and their links to cognitive attributes utilised in decision making in ESRD.

b) Analyse scenarios of the uptake of different dialysis modalities over time and assess the impact on service design (based on system dynamics modeling)

c) Assess impact of centre infrastructure, policy and regulations on implementation dynamics of HHD

d) Conduct an economic evaluation to examine efficiency savings and value

e) Evaluate carer perspectives and burden in the treatment journey of the patient

We believe that such study of HHD uptake, examining barriers and drivers at various levels, using a multi-layered approach that examines patient and organisational factors in parallel using mixed methods (parallel and convergent design) is an ideal methodology to address the research question. A comprehensive study, would aid development of a model of adoption of HHD, which would incorporate variables from both qualitative and quantitative studies. This is the overall aim of the proposed BASIC-HHD study.

## Materials and methods

### Study design

This is a multicentre, prospective, observational cohort study using mixed research methods (combined qualitative and quantitative). Predialysis (CKD-5), incident and prevalent hospital and HHD patients will be studied. The predialysis (CKD-5) cohort will be followed prospectively. A convergent, parallel mixed methods design will be employed to study the cohorts. This means that quantitative and qualitative data will be collected, independent of each other in a single phase, i.e., concurrently. Both quantitative and qualitative datasets will be analysed separately and comparing or combining the results of the quantitative and qualitative analyses will occur at the stage of interpretation.

### Setting

The study is currently underway in the United Kingdom across five centres, in different geographic regions. By design, the centres recruited into the study, have variable prevalence of HHD and categorised to low (<3%), medium (5-8%) and high (>8%) prevalence centres. This heterogeneity provides an important backdrop to the study setting allowing the study of both centre and patient characteristics which might influence the local adoption of this modality. The centres in the study have been chosen on the basis of UKRR information on the HHD prevalence as of June 2010. Several centres also approached the host centre and the final centre participant list for the study was drawn, primarily based on resource availability at the local centres and the size of their home HD/RRT programme.

### Ethical approval

This study has been reviewed and approved by the Greater Manchester West Health Research Authority National Research Ethics Service (NRES) Reference number: 12/NW/0170. The study is on the NIHR (CLRN) portfolio, bearing ID number 12346.

### Study organisation

The BASIC-HHD organisation structure operates from the host centre (MRI). Host centre study team will handle overall management of the study at all centres, through recruitment of research nurses under the supervision of a principal investigator at each one of the participating centres.

**Figure 1:**
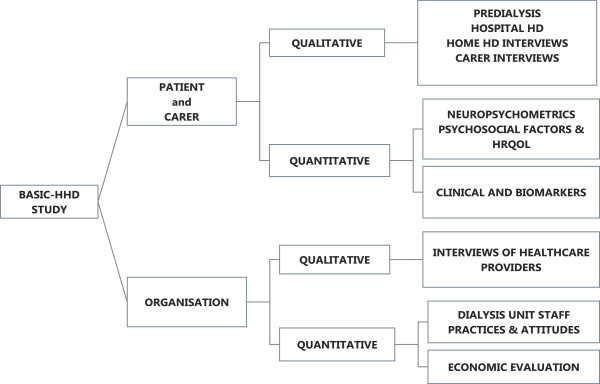
Schematic representation of the scope of the BASIC-HHD study.

The individual centres will manage participant recruitment, data collection and data transfer. The host institution will address protocol education of nurses and colleagues in the participating centres and also help obtain site specific R&D approval prior to commencement of the study (Figure 1).

### Quantitative study arm

#### Inclusion/exclusion criteria

##### Participant inclusion criteria

Participants are eligible for INCLUSION in the study if the following criteria are met-

1. Pre-dialysis patients who are under specialist renal team for management of advanced CKD will be considered for inclusion in the study, from either of these groups-

2. The CKD patient at recruitment, will have eGFR < 10 mls/min (OR) if eGFR between 10-20 mls/min; anticipated dialysis start within 12 months

3. Prevalent conventional HD (in-centre) patients of variable dialysis vintage (minimum time on modality-8 weeks)

4. Prevalent HHD patients of variable dialysis vintage (minimum time on modality-8 weeks)

##### Participant exclusion criteria

Patients will be EXCLUDED from the study if, in the opinion or knowledge of the responsible clinician, any one of the following criteria is present:

1. Dialysis start in a patient not known to the specialist renal team for at least 3 months

2. Life expectancy < 6 months

3. Plans for renal transplantation (Live Donor Transplant) within 6 months of entry into study

4. Inability to complete questionnaires or face-to-face interviews

5. Concomitant major illness limiting assessments and follow-up

6. Factors limiting the offer of home haemodialysis such as uncontrolled psychosis/anxiety, severe learning disability, on-going drug/alcohol abuse, uncontrolled seizure disorder, dementia/poor short term memory.

### Patient participants

Written consent will be obtained from all patients prior to recruitment into the study. Prevalent and incident haemodialysis patients and predialysis patients will be approached for the study.

a. Patients in preparation for RRT, naive to dialysis (COHORT A)

We aim to recruit about 50 pre-dialysis patients from each centre. The anticipated total number of predialysis patients in the study is about 200. Patients will be identified from the prevalent pool (those known to specialist renal team for at least 3 months) and others may need to be recruited prospectively, as they are referred to the advanced chronic kidney disease clinics. To generate a comparator cohort with the reference group (HHD group), some patients recruited will be age (within 5 yrs.) and gender matched with the HHD group (same number as the number of home patients recruited at each centre). The rest of the patient recruitment into this cohort will take into account the diversity in demographics of the presenting ‘pre-dialysis’ population.

b. Patients established in centre HD (COHORT B)

We aim to recruit about 50 pre-dialysis patients from each centre. The anticipated total number of predialysis patients in the study is about 200. Patients will be identified from the prevalent pool (those known to specialist renal team for at least 3 months) and others may need to be recruited prospectively, as they are referred to the advanced chronic kidney disease clinics. To generate a comparator cohort with the reference group (HHD group), some patients recruited will be age (within 5 yrs.) and gender matched with the HHD group (same number as the number of home patients recruited at each centre). The rest of the patient recruitment into this cohort will take into account the diversity in demographics of the presenting ‘pre-dialysis’ population.

c. Patients established on HHD (COHORT C)

From the prevalent and incident HHD pool, variable numbers of patients will be selected from each centre. All HHD patients from the 5 centers will be screened for eligibility. Based on the prevalence rates across all centres, it is expected that about 100 home haemodialysis patients would be able to participate in the study, subject to eligibility.

d. Patient transiting from predialysis to an established dialysis modality COHORT D.

This group is derived from cohort A and will comprise of patients who have started a modality of renal replacement therapy during the 12 months from recruitment. It is anticipated that a third of the patients would have commenced dialysis.

### Duration of subject participation

Individual participants will participate, up to a total of 12 months from recruitment into the study, unless

a. Patient chooses to withdraw from the study

b. Patient develops a major illness within 3 months of study entry that will preclude any assessments or follow-up, necessitating withdrawal from the study

c. Terminal Illness

d. Patient death

Some pre dialysis patients, who may have had to start renal replacement therapy relatively early in the course of the study, will be able to complete participation in the study early.

### Recruitment size and population (Figure 2)

**Figure 2:**
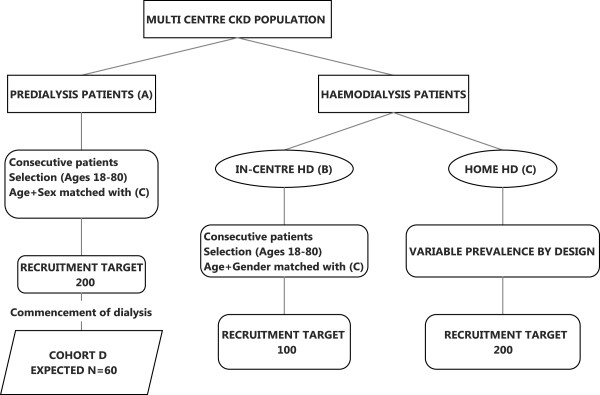
Schema of recruitment size and population.

#### Follow-up visit

All predialysis participants (A) who have given consent to join the study; will be seen after consent, typically when they attend pre-dialysis clinic as per their usual clinic schedule or on a separate day if they so wish. Predialysis patients will also be reviewed >4 months after start of dialysis. During each one of these visits, patients will have blood sampling and neuropsychometric tests. Questionnaires measuring potential psychological predictors of modality choice and adherence will be given on the day and reminders to return the questionnaire will be sent on 2 occasions, one month after handing them out.

All hospital HD (In-centre) patients (B) who have given consent to join the study will have baseline information and laboratory samples collected at the time of their regular dialysis schedules. To standardize the research activity in this cohort, all patients will be seen just before commencement of haemodialysis and all neuropsychometric tests will be carried out mid-week (Wednesday for Mon/Wed/Fri schedule and on a Thursday for a Tue/Thurs/Sat schedule). Blood sampling will be obtained before commencement of dialysis. To ensure a consistent return rate, patients will be requested to complete the questionnaires in hospital, whilst on dialysis and in the first hour of treatment. No further visits will be required.

All home HD patients(C) who have consented to participate in the study will have their baseline information collected in a dedicated clinic for the study, which will be mid-week (Wednesday for Mon/Wed/Fri schedule and on a Thursday for a Tue/Thurs/Sat schedule) and preceding their dialysis schedule by no more than 4 hours. At this time, blood sampling and neuropsychometric tests will be carried out and patients will be requested to complete the questionnaires at home. Time from/to the dialysis session will be documented by the patients. No further visits will be required.

Patients from all cohorts will participate in answering the questionnaires. Non English speaking patients will have the opportunity to answer these queries with the help of an interpreter, using a standard script, as literal translations may not be available in all languages. These questionnaires will be given in a booklet to patients. Dialysis patients will be advised to answer these questions within the first hour of dialysis, so as to avoid the effects of haemodynamic changes on the output. Two attempts will be made to survey the patient cohorts in a 3 month period. These questionnaires will be repeated >3months after commencement of dialysis in the prospectively observed pre-dialysis cohort. All questionnaires will be reviewed for completeness and be manually scored and crosschecked by a second member of the team.

#### Collection of biological samples

**Figure 3:**
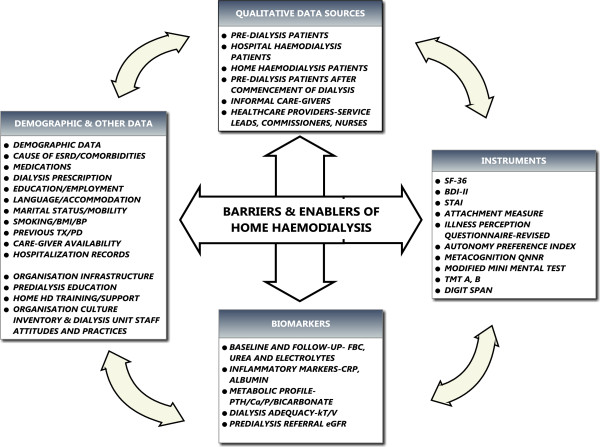
**Synopsis of data to be obtained in the BASIC-HHD study.**

Samples will be sent to the Renal Research Labs for processing and storage. DNA, plasma, cells and a clinical/demographic data set will be held in the laboratories for research, with patient consent. The samples will be obtained from patients at the host centre and frozen in the biobank storage at −80°C, within two hours of collection. This is with a view to study uraemic ‘neuro’toxin assays in these patient cohorts. This remains the subject of a further study relating neurochemistry and behavioural biology. Many of the biochemical parameters being evaluated would be obtained routinely during the course of their medical management and would help in quantifying treatment and disease burden and illness related complications. These samples will be analysed at the South Manchester University Hospital laboratory (Figure 3).

#### Instruments employed in the study and rationale

All participants from the three study cohorts will complete questionnaires based on measures of psychosocial factors which are thought to be predictive of uptake and maintenance of HHD, providing us with a quantitative measure of psychosocial state. The questionnaires have been chosen to obtain information on predictors of outcomes rather than the outcomes only. These combined with interviews will add robustness to the data. The instruments which are being employed assess various aspects of human behaviour, illness perception, state of mind and quality of life.

Healthcare necessarily involves interpersonal contact. This is particularly relevant in end stage renal disease. Clinicians wishing to observe their patients’ interpersonal styles will find descriptive prototypes of adult attachment quite useful. ‘An understanding of interpersonal styles may allow clinicians to adapt medical care to the strengths and vulnerabilities that follow from particular patterns of adult attachment’ [23]. The outcome of this ‘attachment style’ questionnaire will be the first one of its kind in the context of ESRD and the correlation of the questionnaire output with the demands of interpersonal interaction across the modes of dialytic therapies will be made amply clear. Autonomy preference index scale of Ende et al. [24] has been used in this study. It was designed to measure preferences for autonomy in decision making in a general sense, as well as the extent to which people prefer doctors or themselves to make specific management decisions in three clearly defined clinical vignettes. These hypothetical situations have been used as in the original document, without any modification, as it best represents the stable situation, a moderately severe clinical state and a severe episode requiring hospitalisation, which are not specific to ESRD patients, but are unlikely to be unknown to them. The desire to be informed and participate in decision making, will be a desirable attribute in the context of ESRD, and its measurement reflects actual patient preferences. Whether decision making equates to autonomy is a different question and could well be the limitation of this tool.

The SF-36 is a modified version of the Medical Outcomes Study questionnaire. It is a generic instrument with 36 questions, without questions specific to ESRD. It is a reliable and valid tool which has been used in various patient populations, including ESRD [25-27]. There are eight scales describing domains of physical function, social, physical and emotional role function, mental health, bodily pain, vitality and general perception of the state of health.

Depression is particularly unlikely to be recognized in patients with end-stage renal disease (ESRD) because symptoms of depression may overlap with those of uraemia. Consequently, prevalence estimates of depression vary widely from 6% to 34%, depending on the diagnostic instrument and cut off point used [28,29]. The Beck Depression Inventory (BDI), which has a sensitivity of 92% and a specificity of 80% using a cut off of 15 [30] is being used to screen patients for depression, whilst also assessing their cognitive state. The BDI is a self-report inventory that has been extensively validated and used for measuring depression in various population groups, particularly in dialysis patients [31,32]. Although depression in haemodialysis population is well studied, anxiety is also recognized to be a very important problem in dialysis patients. Anxiety may be present independent of other problems or somatised as part of another mental ailment. In this study we have employed a widely used tool for measuring anxiety- Spielberger’s State-trait anxiety Inventory [33]. It clearly differentiates between “state anxiety” and “trait anxiety”. The inventory’s simplicity makes it ideal for evaluating individuals with lower educational backgrounds too.

The Illness Perception Questionnaire-revised (IPQ-R) assesses the following illness perceptions- identity, chronic timeline, cyclical timeline, treatment control, personal control, coherence, causes and emotion reaction. Across several illnesses, the reliability and validity of the IPQ-R has been demonstrated [34]. There is now evidence towards the validity and reliability of the IPQ-R as suitable measure of illness perceptions in the context of ESRD [35,36]. Illness perceptions drive coping and self-management behaviours and hence are an important measure in this study. Home modalities of renal replacement therapy, are very demanding of memory. Beliefs about one’s potential to use memory efficiently will influence self-selection of such therapy [37,38]. Also, individual differences in self-rated memory do not correlate well with objective memory tests- this may imply that people’s beliefs about their memory are inaccurate [39]. This is at least true of the general population. The metacognition questionnaire has two components - the meta-memory and meta-concentration. Additionally, this questionnaire has items worded that are consistently ‘positive’ and avoid a deficit connotation [40]. This has been used in elderly patients, but the questions have no specific age-focus and are applicable to the ESRD population, for the purpose of this study.

To allow for a comprehensive understanding of cognitive function, multiple measures must be included in the neuropsychological test battery. The tests would consider different domains of brain function- psychomotor efficiency and processing speed, learning efficiency and attention. Greater understanding of patient’s cognitive state can be attained through combining subjective cognitive function, for self-reported everyday functioning, in addition to the cognitive psychometric tests. The neuropsychometric tools to be employed are: 3MS/Trail making tests and Digit span test. All neuropsychometric tests and the meta-cognition questionnaire will be conducted in the mid-week, pre-dialysis phase for dialysis patients. This helps standardise the data collection across all centres. All sighted and literate patients will be invited to participate in these tests.

In addition to the outcomes of questionnaire based psychosocial outcomes, any potential association with readily measurable biomarkers could inform day to day clinical management of patients presenting in advanced CKD clinics.

#### Patient study arm- qualitative

For an in-depth exploration of individual perceptions of problems and solutions, semi-structured interviews will be carried out and this process will help define the beliefs, behaviours, attitudes and sensitivities of patients in the different study cohorts. For the qualitative strand, a purposive sampling technique will be employed. The strategy best used in our study is one of maximal variation sampling, such that diverse individuals are chosen who are expected to hold diverse perspectives on the central theme, and their views will reflect a rich and complex picture of the reality. The qualitative idea is not to generalize from the sample but to obtain an in depth understanding of the issues in a few people who have been sampled, unlike the quantitative study arm.

The study sample will comprise pre-dialysis patients (Cohorts A + D), home haemodialysis patients (Cohort C) and hospital haemodialysis patients (cohort B). All adult patients aged 18 and over, with end stage renal diseases, who meet the inclusion criteria and no exclusion criteria, will be considered eligible for the study. The aim is to conduct about 20 interviews in each cohort or until thematic saturation is attained.

A recruitment grid is designed taking into consideration three factors- age, gender and ethnicity. Although it is not a requirement for recruitment, every effort will be taken to include patients with varying comorbidity profile. English, Hindi and Urdu speaking patients would be considered, representing the local population demographics.

Participants will be approached at their regular clinic consults by researchers with the study information leaflet and consent form. Where the patient expresses a willingness to consider participation, telephone contact will be made after a minimum of 24 hours of providing information. At this time, any questions will be answered and if the participant remained willing, an appointment for the interview will be scheduled. In a majority of instances this would be in the patient’s own home or at the patient’s request, the venue would include the hospital. Suitably qualified individuals will carry out the interviews. All interviews will be one-to-one. They will be audio-taped and then transcribed verbatim.

For purposes of the study, an interview schedule or topic guide has been developed with a view to cover the following areas-

a. Barriers and enablers of home haemodialysis as patients perceive them

b. The potential solutions as seen by patients

c. Impact of self-cannulation on decision-making

d. Views on assisted home haemodialysis

### Carer study – qualitative

#### Participant eligibility (carers)

All adult carers (aged 18 and over) of patients undertaking home haemodialysis or carers of patients who are in the decision-making process, who meet the inclusion criteria and none of the exclusion criteria will be considered eligible for this study.

#### Carer Inclusion criteria

Participants are eligible for INCLUSION in the study if the following criteria are met-

–The patient needs to consider the individual to be their carer.

–The individual self-defines themselves as the patient’s carer.

For the purposes of this research, a broad definition of the carer’s role is employed. A carer in the context of home haemodialysis is understood to range from providing emotional support to the patient, to taking a degree of responsibility for the patient’s dialysis procedure (Blogg and Hyde, [1]).

#### Carer exclusion criteria

Participants will be EXCLUDED from the study if, in the opinion or knowledge of the interviewer, either of these criteria is present:

–An established primary care diagnosis of psychiatric illness

–A life-threatening physical illness

The participant information sheet will be provided to the participant, to make an informed decision concerning in the study. A copy of the signed informed consent and information sheet will be given to the carer. Carers will undertake semi-structured interviews, at a place of their choosing- their homes or at the hospital. About 20 carers will be interviewed or until thematic saturation is attained. All interviews will be audio-taped and transcribed verbatim. Adequate steps will be undertaken to ensure that distress, if any, caused to them when they verbalize their fears will be dealt with appropriately and professionally.

### Provider study arm – qualitative

Alongside patient assessments, investigators will obtain information pertaining to the centres offering home haemodialysis. More specifically, this study seeks to help policymakers and renal care providers to understand and overcome barriers to delivering complex, patient-led medical procedures in the home by addressing the organizational, financial and policy influences on the uptake of HHD. This research project investigates how regulation, reimbursement rules and health policy impacts the provision of care for patients with chronic kidney disease.

Specific questions which will be posed include-

1. What factors impact on the adoption of technological innovations in renal care, especially home haemodialysis?

2. How do factors such as cost and financial arrangements (e.g. payment, incentives or penalties), implementation (e.g. training), intra-organisational issues (e.g. clinical leadership, resistance of incumbents to innovation), or environmental context (e.g. space at home, distance between home and clinic) limit the use of home haemodialysis to a level far below of what is generally considered desirable?

3. What impact do financial regulatory changes (e.g. PbR and the ‘best tariff practice’ in the UK, bundling of services and changes to reimbursement rules for home haemodialysis in the US) have on the behaviour of healthcare professionals and on care provision?

4. What impact will different policy choices and payment regulations likely have on the use of different dialysis modalities in the future?

#### Research approach

This study will combine qualitative research through interviews with modeling. The interviews will address research questions 1, 2 and 3 and modeling will address question 4.

#### Design

This is a comparative multi-centre study. It will employ qualitative methodology in the form of 45 to 60 minutes in-depth semi-structured interviews. Data from the qualitative case studies and background interviews will be used to inform simulation modeling exercises to investigate the implications of implementing HHD under differing policy and financing options, and with differing care provision models.

#### Respondents

The background interviews will involve selected participants from leading (i) dialysis service organizations, (ii) dialysis machine design and manufacturing firms, (iii) national renal policy-making bodies.

The case studies interviews will involve (i) healthcare professionals, (ii) managers of selected renal care centers. The aim is to interview all personnel involved in the modality decision making process as well as the care of renal patients in each centre. These include:

•Physicians

•Nurses

•Medical/Clinical directors

•Commissioners

## Economic evaluation

Although the study is not powered to evaluate a definitive economic benefit of HHD, we will estimate costs related to hospitalizations, medications and cost-utility from the provider’s perspective based on data from at least 2 case-study sites. The study will analyse the incremental costs of providing HHD, including direct costs (disposables, equipment, personnel, training, monitoring and technical services) and in-centre costs (HD unit overhead). We will also explore the potential impact of policy choices and financial conditions on HHD implementation under a variety of scenarios. We will use simulation modeling to analyse such impacts on patient flows through the system, the choice of dialysis modality and costs.

### Provider study arm-quantitative

From the different centres participating in the study, centre specific information will be obtained. The broad areas where detailed information will be sought include-

a. Infrastructure

b. Predialysis education and preparation

c. Training for HHD

d. Post-Discharge support structure

#### Dialysis unit staff-attitudes and practices (Organizational Culture Inventory®)

Understanding the collective thought processes informing a certain type of practice or behaviour is fundamental to effecting the desired change. The use of a quantitative instrument together with ethnographic approach followed by triangulation of results is likely to give a more complete picture of the practice in a unit. Such an approach in an area like dialysis methods has not been undertaken before and would add value to the information available. ‘The main appeal of such a method, lies in seeking to engage organizations and their problems on the level of meaning’ [41]. One may not assume that everyone has a similar understanding of the issues and may have opinions and judgments that vary from the perceived norm.

This Inventory by Cooke and Lafferty, is a quantitative measure of the culture in the unit/organization [42]. It evaluates the shared norms and expectations that guide thinking and behaviour of group members. It has previously been employed in the healthcare sector. It has 120 pre-defined questions and will have 20 additional questions introduced specific to the study context. The responses are made in a 5 point Likert scale. It is likely to take up to 20–25 minutes for completion. The tool has good face validity, and strong psychometric underpinning. Results can be graphically illustrated. The problems may be encountered with the length of the tool, but sufficient time will be given to staff to engage with this tool, as we investigate its application in a novel context.

### Data protection

Each patient enrolled into the study, is assigned a unique identification number. All personal identification data are stored in hospital computers only and separated from medical and research data. Biological samples are identifiable only with their unique sample identifier and no patient identifiers are available within the Biobank. All information pertaining to OCI® from specific centres are only known to the lead researcher and not available to the organisation that holds the license to conduct the survey.

### Quality assurance

In order to ensure consistent performance across all centres, single researcher (AJ) has conducted induction days across all centres. The database will be continually cleaned and examined to ensure data entry is accurate and missing data is minimised. The database is backed up regularly to ensure no loss of data. Biological samples obtained are collected, processed and stored in appropriate conditions and by certified personnel. All qualitative aspects of the study will be reviewed by at least two qualified researchers. All costs related information will be procured after checking relevant documents from appropriate authorities.

### Enrolment to-date

Enrolment as of the end of April 2013 comprises a total number of 350 patients across five centres. It is anticipated that the recruitment period will end in late 2013. Recruitment for the qualitative aspects of the study is about 75% complete.

### Data analyses and statistical considerations

#### Quantitative data analysis

The sample size of the BASIC-HHD study of 500 patients was chosen as a realistically attainable cohort size based on feasibility considerations. This sample size ensures adequate statistical power to differentiate cohorts showing different behaviours with respect to the primary and secondary objectives. The power analysis is also based on the effects expected in the clinical measures.

Demographic data will be reported using means and standard deviations (for normally distributed data) or median and interquartile/full ranges (for skewed data) where appropriate. Simple comparisons between baseline data from the three study cohorts will be made using two-sided tests; analyses of variance for normally distributed data, Kruskal-Wallis for non-normal continuous data and ordinal data, and chi-square tests for categorical data, with the conventional 5% significance level. Individual cognitive test scores will be reported as mean and standard deviations. Scores will be reported both as normalized scores. In normalized scores, data are fit to a normalized scale allowing direct comparisons across scores. Factors which influence uptake and maintenance will be identified using multiple logistic regression analysis of the pre-dialysis cohort. Comparisons between the HHD (C) and Hospital HD (B) cohorts will use t-tests, Mann–Whitney U-tests and chi-square tests as appropriate, followed by multiple logistic regression analysis to identify significant independent discriminatory factors. The study will have 80% power to detect differences in the percentage of patients having particular factor attributes of 19% or more between the age-sex matched cohorts of 100 patients (i.e. 20% vs. 39%, equivalent to an odds ratio of approximately 2.5). For the pre-dialysis patients, of whom 10% are estimated to take up home haemodialysis, the study will have 80% power to detect prognostic factors with an odds ratio of 4 or more for the whole cohort of 200 patients.

#### Qualitative data analysis

The interviews will be analyzed using thematic analysis, a methodologically and epistemologically flexible approach; it is partly guided by the aims and the research questions stated at the beginning of the project and partly guided by the researcher’s active identification of themes based on the accounts of participants’ own views and experience. At least two researchers will analyze the interviews independently ensuring the reliability of the analyses. The initial coding of each interview will be compiled by both researchers independently, and the coding frames and themes will be refined and elaborated collectively in a dynamic way as more data gets collected. As sequential analysis progresses, significant data will be compressed so as to adhere around several analytic schema. In order to ensure the reliability of the qualitative analysis, the application of the coding frame by independent researchers will be periodically cross checked to establish a degree of uniformity.

Several techniques will be used to ensure the validity of the qualitative analysis including:

•Respondent validation

•Triangulation: (by looking for commonalities and anomalies at a sample level)

•Fair Dealing: our theoretical sampling approach explicitly aims to incorporate a wide range of different perspectives

#### Simulation modeling analysis

Using national and international renal patient registry data in addition to our findings from interviews and our analysis of the literature, we will also apply scenario ("what … if") analysis using system dynamics modeling to investigate the impact of different policy choices and financial models on dialysis technology adoption, care pathways and costs. This will allow us to explore the factors influencing the choice of dialysis modalities and develop a number of scenarios of the likely development under a range of policy options (including changes in reimbursement/funding rules). The modeling explore the role of the factors identified as barrier and drivers in the case studies and also draw on the literature review, the data collected in the background interviews with industrialists, policy makers and healthcare professionals, historical registry data and policy documents.

### Integration of qualitative and quantitative data and study outcomes

Combined qualitative and quantitative research methods are finding increased acceptance in clinical and biomedical arenas. The choice of a research methodology is typically informed by a research strategy. The study of adoption barriers to uptake of home haemodialysis would ideally involve research in clinical and biological factors interacting with psychosocial influences on health of the individuals concerned and health services delivery research simultaneously. It is also important to understand these together as there is little point in developing services or measuring patients’ outcome of health care, without an understanding of how people’s beliefs and expectations about health, illness and treatment regimens on offer may interact with those of health professionals, and thereby influence uptake of services and adherence to therapy.

The choice of methodology to evaluate our research question is one of mixed methods. This choice evolves from a ‘pragmatist’ world-view which embraces paradigms that influence and underlie the conduct of qualitative and quantitative research methods, through a social science theoretical lens. The BASIC-HHD study will adopt a dynamic, synergistic approach [43] to the design process. This implies that, the sum of quantitative and qualitative research is greater than either approach alone.

#### Rationale for mixing methods

Bryman, in his work, offered 16 different reasons for why one may choose to mix methods in research [44]. In our study, multiple reasons may be cited and new reasons may emerge as the study is underway. The following tabulation of typology of reasons (adapted from Bryman) helps understand the purpose of the methodology (Table 1).

**Table 1:** Typology of reasons for mixing methods

**Terminology**	**Explanation**
Triangulation	Qualitative and Quantitative methods might be combined so as to mutually corroborate the findings
Offset	The methods have their own strengths and weaknesses, so combining the two would allow to offset the weaknesses and draw on strengths of both
Completeness	A more comprehensive account of the research question is possible
Different research questions	The two methods can answer different research questions
Explanation	The findings of one method may be used to explain the findings of the other
Credibility	Employing both approaches enhances the integrity of the findings

#### The four key decisions in the choice of design

These decisions address the different ways in which the quantitative and qualitative strands relate to each other. The strand refers to the component of the study that encompasses the basic process of conducting qualitative or quantitative research: the question, data collection, data analysis and interpretation of results [45].

a. The level of interaction between the qualitative and quantitative strands: In our study, the implementation of the two strands will be independent of each other, i.e., the data collection and analysis will be separate, and the two will be mixed when drawing overall conclusions at the end of the study.

b. The priority of the quantitative and qualitative strands: The two strands will have equal emphasis i.e., both will have an equally important role in addressing the research question.

c. The timing of the quantitative and qualitative strands: The timing of the two strands will be concurrent, i.e., both methods will be employed in a single phase.

d. Mixing the strands: Mixing also referred to as, combining and integrating, is the explicit interrelating of the two strands, and this point of interface during our study, will be at the stage of interpretation of the results of data analysis. Primary mixing strategy is- merger after separate data analysis.

Crystallisation of findings from both components will be reported when discussing the results of the study. There is scope for comparison of raw datasets of one individual from both methods, as an example, the interview transcript and questionnaire reports can be compared and patterns looked for across cases. The notation system for this mixed methods design is QUAN + QUAL, i.e., both methods occur concurrently [46].

#### Study outcomes

The quantitative and qualitative studies which are being undertaken to understand patient clinical and psychosocial parameters, in the context of healthcare infrastructure and provider views from geographically distinct sites, will give the breadth and depth of problem perception and solutions. We seek to identify systemic issues that may deter the uptake of HHD and understand factors which may define the atlas of variation, to develop a tool to implement a practice changing model of care. Additionally, the study will highlight the beliefs and concerns of the major stakeholders- patients, care givers and healthcare providers on HHD. We will be able to ascertain the range of interventions and assistance that may be necessary for successful adoption. The association (if any) between patient-psychosocial factors and biochemical parameters, including specific toxin assays in the context of ESRD will also be analysed. The study findings will be disseminated to clinicians, organisations and health care strategists as guidance to inform future policy. Lessons from the implementation of the study design would also pave way for more holistic research of chronic diseases in health care systems using this methodology.

## Discussion

Over the last five decades, the growth in demand for dialysis has increased exponentially. There is a growing need to develop methods for improving treatment outcomes whilst paying attention to costs. It is in this context, that there is renewed interest in home haemodialysis. The proposed benefits in HHD offering extended dialysis schedules are supported by randomised controlled trials and several observational studies. Despite the rhetorical remarks on the benefits of HHD, its practical uptake has been somewhat slow over the last decade and steps are being taken to increase its adoption, in the UK and globally. In the study of adoption of a well understood, complex intervention, such as home haemodialysis, a robust study methodology is important to delineate the issues facing the patients, caregivers and healthcare providers. Partnerships will be required between all stakeholders to adopt changes in attitudes, with the necessary regulatory alterations to implement clinically superior, patient-focused dialysis treatment programs, where informed patient choice is paramount.

Methodology is the rationale and philosophical assumption underlying a particular study and not merely, a collection of methods, although, methodology leads to and informs methods. Mixed methods papers in the field of nephrology are very few [47]. Historically, studies have been done to understand some aspects of this rather large question on ‘barriers and enablers of home haemodialysis’. Reports on different aspects of this issue, come from study designs which do not automatically lend themselves to accuracy or detail. These include close-ended questionnaire based surveys, completed probably, by individuals who are driven enthusiasts propagating home haemodialysis, thereby introducing bias. In some instances, home dialysis incorporating peritoneal dialysis and home haemodialysis, have been studied and although, this would capture the notion of ‘self-care’, nuances, specific to home haemodialysis may not be elucidated in detail. Identifying the barriers and enablers of home HD is only meaningful when probable solutions to the issues projected from studies, are also provided. In addition, within the financial constraints of the current health service provision, cost models and understanding optimal service delivery designs are fundamental to effecting the desirable change.

The BASIC-HHD study has been designed to understand the patient, the care-giver and the healthcare provider in a rapidly changing health care climate, where emphasis on patient-centred choice and ‘care closer to home’ remains at the core of NHS ethos. The strength of this study lies in its methodology wherein a complex intervention of a life sustaining self-care technology is systematically studied in a holistic sense incorporating qualitative and quantitative research components studied simultaneously with equal emphasis in centres with varying prevalence and uptake of HHD. Incorporating multiple research methods will help acquire a three dimensional view of the research findings, more likely to yield lasting solutions to the research questions.

Health systems reforms, have in the last 20 years evolved from provision of structured to managed care as, structural changes, on their own may not be able to deliver anticipated improvements in quality and performance in health care [48]. This has not been studied in a smaller context such as dialysis provision centres. Organisational culture denotes much more than just the way things are done and is a unique aspect of this study arm. The study will analyse attitudes and practices in the varying dialysis units. This may help distinguish frontline service delivery (functional behaviour) from organizational (structural) influences.

The BASIC HHD is a pragmatic study, suitably equipped with requisite expertise to carry out the work. We have invested the effort and time to form a team of researchers who are keen to study several facets of the research question in parallel, from across UK. Prospective recruitment of the pre-dialysis cohort will help understand the patient journey better and we anticipate recruiting patients from across the spectrum of illness severity. This will improve the generalizability of our findings. Bias and random error in handling data will be minimised by validation checks by the researcher on data entry by research nurses. Periodic visits to the participating units will ensure uninterrupted and uniform data gathering, and an opportunity to troubleshoot problems as they arise. Conceptually, the BASIC-HHD study is intended to be both confirmatory and exploratory in design and provide a scaffold for ancillary studies addressing specific psychosocial characteristics and biomarkers in the different study cohorts. Planned studies include an in-depth characterization of uraemic ‘neuro’toxins, namely the guanidino compounds and their impact on cognitive outcomes and decision making processes in ESRD. Exploratory work in search of a candidate biomarker in biological samples (Blood spots and saliva) for cognitive dysfunction in the context of ESRD is underway. It is also likely that progression of the ‘uraemic state’ impacts on physical factors such as, global DNA methylation, gene expression and metabolic pathways, which may influence mental health and quality of life. Its associations with psychological outcomes can now be combined to generate valuable information, for patient care.

In conclusion, the BASIC-HHD is a unique study in dialysis medicine, which will assemble pivotal information on dialysis modality choice and uptake, investigating users, caregivers and care delivery processes and study their variation in a multi-layered analytical approach within a single health care system. The study results would define modality specific service and patient pathway redesign with the potential of a paradigm shift in practice and providing future directions in dialysis care.

## Competing interests

The authors declare that they have no competing interests.

## Authors’ contributions

All authors have contributed to the overall design of this study. SM is the chief investigator with overall responsibility for the BASIC-HHD study. AJ, as the study lead, participated in the study design and coordination, prepared the protocol, obtained ethical approval and participated in the liaising with all participating units. AW is responsible for overseeing the qualitative components of the patient study arm and the ICBS team (JB, SB and IA) developed the organisation qualitative study arm and the health economics analysis plan. JM is the study statistician. PB is director of the renal research laboratory at Manchester Royal Infirmary and is part of the study steering group. All authors have read and approved the final manuscript.

## Pre-publication history

The pre-publication history for this paper can be accessed here:

http://www.biomedcentral.com/1471-2369/14/197/prepub
